# Potential terahertz therapeutic strategy for the prevention or mitigation of Alzheimer’s disease pathology

**DOI:** 10.1038/s41377-023-01289-x

**Published:** 2023-10-18

**Authors:** Xinke Wang, Yan Zhang

**Affiliations:** Beijing Key Laboratory of Metamaterials and Devices, Key Laboratory of Terahertz Optoelectronics, Ministry of Education, Beijing Advanced Innovation Center for Imaging Theory and Technology, Department of Physics, Capital Normal University, 100048 Beijing, China

**Keywords:** Biophotonics, Terahertz optics

## Abstract

With terahertz irradiation with a specific frequency, the fibrotic progression of β-amyloid oligomers is suppressed, which provides a potential therapeutic strategy for Alzheimer’s disease.

Because the vibration and rotation energy levels of numerous biomacromolecules lie within the terahertz (THz) frequency band, THz technology is always considered a promising tool for biomedical applications^1,2^. In previous reports, the researchers preferred to use THz sensing and imaging techniques for biomedical diagnosis. By comparing THz spectra of normal and abnormal tissues, the levels of liver injury and burn severity can be accurately differentiated^3,4^. By analyzing THz images of tissue slices, the regions of brain tumors and the metastatic states of lymph nodes can be exactly discriminated^5–7^. By observing THz birefringence phenomenon in enamel, healthy teeth and dental caries can be clearly identified^8^. In recent research, the interactions between THz radiation and tissues are strengthened by the introduction of metamaterials and the sensitivities of THz systems are enhanced for biomedical diagnosis^9,10^. These works positively prompt the development of THz biomedical technology.

With the performance enhancements of THz radiation sources and associated techniques^11,12^, the researchers gradually recognize that some physiological processes can be directly intervened by THz wave, such as the acceleration of DNA unwinding^13^ and the permeability enhancement of the voltage-gated calcium channel^14^. It means that THz technology may be directly applied in the treatment of pathological tissues, which undoubtedly exploits the possibilities of THz biomedical applications.

In this issue of eLight, W. Peng and her collaborators from Air Force Medical University, National Innovation Institute of Defense Technology, University of Shanghai for Science and Technology, and Peking University present a positive effect of THz radiation for delaying the fibrotic progression of β-amyloid (Aβ) oligomers^15^. After being exposed to THz radiation with a 34.88 THz central frequency, Aβ in the dimethyl sulfoxide solvent shows a lower ThT fluorescence intensity compared to the reference sample without THz irradiation. By comparing Fourier transform infrared spectra of the THz-loaded and reference samples, it can be seen that the percentage content of the β-sheet stack is significantly reduced and the fibrotic progression is slowed down in 96 h after THz irradiation. In addition, the temperature shift of the THz-loaded sample is also monitored, which exhibits that the THz wave has no strong thermal effect on proteins. Moreover, it has been demonstrated that THz wave is harmless to normal cells by detecting the cell viability and mitochondrial membrane potential. Through a long-time molecular dynamic (MD) simulation, the interaction mechanism between the THz wave and Aβ oligomer is revealed. The structure of the β-sheet is transformed to more coil and bend form by the THz wave. In the meantime, the effect also shows a pronounced frequency-dependent characteristic. Namely, the protein system resonates with the electromagnetic wave with a specific frequency and further induces the dissociation force.

Overall, because amyloid deposition is a main hallmark for neurodegenerative diseases, the work indicates that THz technology can be utilized as a therapeutic strategy to regulate the conformations of pathological proteins and mitigate Alzheimer’s disease, as shown in Fig. 1. Of course, there is much improvement room for the practical application of this technique, such as the deeper understanding of the intervention mechanism, the upgrade of the curative effect, and the optimization of the THz system. Nevertheless, it can be anticipated that THz technology will contribute to the biomedical field as an optical therapeutic means.

**Fig. 1:**
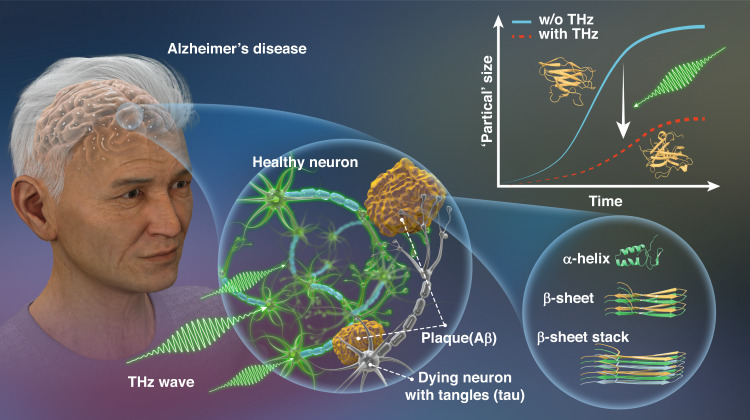
The presence of extracellular β-amyloid deposition remains the primary neuropathologic feature for Alzheimer’s disease diagnosis. The amyloid deposition begins with the conversion of native monomer with more α-helix to an alternative conformation with β-sheet that self-associates into ordered assemblies. The terahertz wave at the frequency of 34.88 THz can delay the fibrosis dynamic curve

## References

[1] Peng Y (2020). Terahertz spectroscopy in biomedical field: a review on signal-to-noise ratio improvement. PhotoniX.

[2] Peng Y (2021). Three-step one-way model in terahertz biomedical detection. PhotoniX.

[3] Osman OB (2020). Differentiation of burn wounds in an in vivo porcine model using terahertz spectroscopy. Biomed. Opt. Express.

[4] Huang PJ (2019). Analysis and inspection techniques for mouse liver injury based on terahertz spectroscopy. Opt. Express.

[5] Oh SJ (2014). Study of freshly excised brain tissues using terahertz imaging. Biomed. Opt. Express.

[6] Yamaguchi S (2016). Brain tumor imaging of rat fresh tissue using terahertz spectroscopy. Sci. Rep..

[7] Park JY (2017). Terahertz imaging of metastatic lymph nodes using spectroscopic integration technique. Biomed. Opt. Express.

[8] Cai JH (2022). Dental caries diagnosis using terahertz spectroscopy and birefringence. Opt. Express.

[9] Ha T (2022). Subwavelength terahertz resonance imaging (STRING) for molecular fingerprinting. Nano Lett..

[10] Lee SH (2020). Label-free brain tissue imaging using large-area terahertz metamaterials. Biosens. Bioelectron..

[11] Khalatpour A (2021). High-power portable terahertz laser systems. Nat. Photonics.

[12] Liang YF (2023). Widely tunable electron bunch trains for the generation of high-power narrowband 1-10 THz radiation. Nat. Photonics.

[13] Wu KJ (2020). Terahertz wave accelerates DNA unwinding: a molecular dynamics simulation study. J. Phys. Chem. Lett..

[14] Li YM (2021). Terahertz wave enhances permeability of the voltage-gated calcium channel. J. Am. Chem. Soc..

[15] Peng WY (2023). High-frequency terahertz waves disrupt Alzheimer’s β-amyloid fibril formation. eLight.

